# Supplementation of a western diet with golden kiwifruits (*Actinidia chinensis var*.'Hort 16A':) effects on biomarkers of oxidation damage and antioxidant protection

**DOI:** 10.1186/1475-2891-10-54

**Published:** 2011-05-18

**Authors:** Asgeir Brevik, Isabel Gaivão, Tirill Medin, Aud Jørgenesen, Anita Piasek, Johanna Elilasson, Anette Karlsen, Rune Blomhoff, Turid Veggan, Asim K Duttaroy, Andrew R Collins

**Affiliations:** 1 Department of Chemical Toxicology, Division of Environmental Medicine, Norwegian Institute of Public Health, P.O.Box 4404 Nydalen, N-0403 OSLO, Norway; 2 Genetic and Biotechnology Department, University of Trás-os-Montes and Alto Douro, Quinta de Prados, 5001 Vila Real Codex, Portugal; 3 Department of Nutrition, Faculty of Medicine, University of Oslo, PB 1046 Blindern, 0316 Oslo, Norway; 4 Gdansk University of Technology, Gdansk, Poland; 5 TATAA Biocenter AB, Odinsgatan 28, 411 03 Göteborg, Sweden

## Abstract

**Background:**

The health positive effects of diets high in fruits and vegetables are generally not replicated in supplementation trials with isolated antioxidants and vitamins, and as a consequence the emphasis of chronic disease prevention has shifted to whole foods and whole food products.

**Methods:**

We carried out a human intervention trial with the golden kiwifruit, Actinidia chinensis, measuring markers of antioxidant status, DNA stability, plasma lipids, and platelet aggregation. Our hypothesis was that supplementation of a normal diet with kiwifruits would have an effect on biomarkers of oxidative status. Healthy volunteers supplemented a normal diet with either one or two golden kiwifruits per day in a cross-over study lasting 2 × 4 weeks. Plasma levels of vitamin C, and carotenoids, and the ferric reducing activity of plasma (FRAP) were measured. Malondialdehyde was assessed as a biomarker of lipid oxidation. Effects on DNA damage in circulating lymphocytes were estimated using the comet assay with enzyme modification to measure specific lesions; another modification allowed estimation of DNA repair.

**Results:**

Plasma vitamin C increased after supplementation as did resistance towards H_2_O_2_-induced DNA damage. Purine oxidation in lymphocyte DNA decreased significantly after one kiwifruit per day, pyrimidine oxidation decreased after two fruits per day. Neither DNA base excision nor nucleotide excision repair was influenced by kiwifruit consumption. Malondialdehyde was not affected, but plasma triglycerides decreased. Whole blood platelet aggregation was decreased by kiwifruit supplementation.

**Conclusion:**

Golden kiwifruit consumption strengthens resistance towards endogenous oxidative damage.

## Introduction

Diets rich in fruits and vegetables offer protection against the development of cardiovascular diseases (CVD), diabetes and cancer [1-3]. A common factor in the aetiology of these diseases seems to be damage to biomolecules caused by reactive oxygen species. Powerful endogenous antioxidant defences are thought to be augmented by dietary antioxidants, and so much of the protective effect of fruits and vegetables has been attributed to their high content of antioxidants [4,5]. However, attempts to boost human resistance to cardiovascular disease and cancer through supplementation trials with isolated antioxidants and vitamins have proved disappointing [6-8], and there is no reason to believe that negative effects of unhealthy diets and lifestyle can be remedied through the use of antioxidant supplements. Hence, the emphasis of chronic disease prevention policy has shifted to whole foods and whole food products. In addition to recognised antioxidants such as vitamins C and E, carotenoids and flavonoids, fruits and vegetables contain innumerable other phytochemicals, with known or (mostly) unknown effects on human metabolism. Antioxidant activity is clearly not the whole story.

Kiwifruit is particularly rich in vitamin C (ascorbic acid), but also contains a wide range of other phytochemicals. The common green kiwifruit, *Actinidia deliciosa*, has been used as a 'model' fruit in several trials to examine effects on biomarkers relevant to both cancer and CVD. Typically green kiwifruit contains approximately 85 mg vitamin C per 100 mg fresh weight [9]. A kiwifruit extract has powerful antioxidant activity *in vitro *[10], and in humans, regular consumption of this fruit inhibits platelet aggregation [11], decreases endogenous oxidation of lymphocyte DNA, protects lymphocyte DNA from oxidation *in vitro*, and enhances the capacity of lymphocytes to repair DNA oxidation damage [10,12-15]. The more recently available 'golden' kiwifruit *Actinidia chinensis *var. Hort 16A, differs significantly in phytochemical make-up (with 20% higher vitamin C content [9]), demonstrating higher FRAP values [16] than the green kiwifruit. Based on these properties the golden kiwifruit would be expected to show stronger protection against effects of oxidative damage *in-vivo*. To test this hypothesis, we conducted a human dietary intervention trial with golden kiwifruit, examining potential effects on platelet function, plasma antioxidant status, DNA oxidation, and base excision repair (BER), as well as nucleotide excision repair (NER) activity. Plasma malondialdehyde (MDA) was measured by HPLC. As a product of lipid peroxidation, it acts as a marker for overall oxidative stress.

Our results indicate that golden kiwifruit strengthens our resistance towards endogenous oxidative damage, but our results do not support the view that the golden kiwifruit provides noticeably stronger protection against oxidative damage than the green variety.

## Experimental Methods

### Study design and participants

Twenty-four men and women (20-57 years, BMI 20-30 kg/m²) were recruited from the university and neighbouring companies through poster advertising and email campaigns. After a screening interview, subjects eating modest amounts of fruits and vegetables were selected. Exclusion criteria were: use of contraceptive pills, medicines or supplements; diets aimed at weight correction; diagnosed diabetes, cancer or cardiovascular disease; habitual consumption of >30 units of alcohol/week (15 glasses of wine); habitual undertaking of >6h vigorous exercise/week (assessed by exercise questionnaires completed at screening session); abnormal menstrual cycle/hormone replacement therapy; or a high intake of fruits and vegetables (> 450 grams per day). The study was approved by the regional committee of ethics in medical science. Written consent was obtained from all participants.

Subjects received two different 'doses' of kiwifruit in two 4-week supplementation periods separated by a 4-week washout period. The cross-over design of the study minimises so-called seasonal effects. Subjects were randomized into two groups taking the kiwifruit doses in different order; either one kiwifruit per day in the first period, two in the second, or two per day in the first period, one in the second. Subjects were not given specific times at which to consume the fruit, but were asked to add the fruit to their normal diet. They were specifically instructed to avoid increasing their habitual low to moderate intake of fruits and vegetables.

### Blood sampling

Four venous blood samples were taken from overnight-fasted subjects: 1) pre-intervention, first period; 2) post-intervention, first period; 3) pre-intervention, second period; 4) post-intervention, second period. A pre-study run-in sample was taken to practice sampling routines. A post-study sample was taken to check for return to baseline levels 4 weeks after the end of supplementation.

Blood was collected in Cell Preparation Tubes (Beckton Dickinson) and centrifuged (700 × g, 10 min at 4°C) to separate plasma, red cells and mononuclear cells. Plasma aliquots were snap-frozen in liquid nitrogen and stored at -80°C; those intended for vitamin C analysis were acidified with an equal volume of 10% meta-phosphoric acid (MPA). The MPA solution was freshly prepared (within 14 days) and stored at 4°C. Mononuclear cells were analysed for DNA damage on the day of isolation. Mononuclear cells suspended in 45 mM HEPES, 0.4 M KCl, 1 mM EDTA, 0.1 mM dithiothreitol, 10% glycerol, pH7.8 at 5 × 10^7 ^cells per ml were snap-frozen in liquid nitrogen and stored at -80°C for use in the *in vitro *BER and NER assays.

### Measurement of plasma vitamin C

The frozen, acidified samples were thawed, and centrifuged (3500 × g) at +4°C for 10 min. Following the sentrifugation of the acidified heparin plasma, 100 μL of the clear supernatant was diluted with 400 μL of the mobile phase (2% acetonitrile in 2.5 mM NaH_2_PO4, 2.5 mM dodecyltrimethyl ammonium chloride and 1.25 mM Na_2_EDTA in Milli-Q water) for direct determination of ascorbic acid (AA). For the determination of total ascorbic acid (TAA), 100 uL of the clear supernatant was subjected to reduction for 7 min with the addition of 50 μL 2.3 mmol/L TCEP in 800 mmol/L trizmabuffer (pH 9.0). Subsequently, 350 μL of the mobile phase was added. For separation of ascorbic acid from interfering plasma constituents, a Chromolith Performance RP18-e, 4.6 mm × 100 mm column was used, with a Chromolith Performance RP18-e, 4.6 mm × 10 mm guard column. The injection volume used was 5 μL and the flow rate was 6.0 mL/min. A variable wavelength UV detector was used at 264 nm. A detailed protocol has been published [17].

### Measurement of plasma carotenoids

The carotenoids lutein, zeaxanthin, β-kryptoxanthin, α-carotene, β-carotene and lycopene were determined in plasma by HPLC. Proteins were precipitated and removed by the addition of a 450 μL isopropanol to 100 μL plasma, followed by centrifugation at 3.000 g at 4°C for 15 min. The internal standard astaxanthin was added with the isopropanol. Twenty five μL of the clear supernatant were used for analysis. The mobile phases consisted of A: 20% water and 24% acetone in ethanol and B: acetone. The gradient conditions were as follows: From 2 to 100% mobile phase B within 20 min, followed by 100% mobile phase B for 15 min. Detection was performed at 453 nm using a variable wavelength detector. Plasma calibrators quantified against the NIST 968c SRM were used as standards.

### FRAP assay

Ferric Reducing Activity of Plasma (FRAP) was determined as described [18]. The modified assay measures FRAP after removal of uric acid and proteins from the plasma and more accurately indicates the contribution of dietary antioxidants. Briefly, 30 μl uricase (0.1 unit/10 μl in Tris buffer, pH 8.5) was added to 60 μl plasma at room temperature. The samples were incubated for 6 min. 120 μl absolute ethanol was added and the samples were vortex-mixed. After incubation for 5 min at 4°C to allow complete protein precipitation, the samples were centrifuged at 13,000 × g for 5 min. The supernatant was used for FRAP analysis as described [18].

### Measurement of malondialdehyde in plasma

Plasma samples were diluted ten-fold in a final reaction mix containing 0.8% SDS, 10% acetic acid, and 0.17% thiobarbituric acid, incubated for 1 h at 100°C, centrifuged, and then injected (50 μL) onto a Chromolith Performance RP 18e 10 cm (internal diameter: 3 cm) column (Merck) connected to a Brownlee pre-column (RP-18, 5u, OD-GU, 30 × 4,6 mm); the mobile phase was a 6:4 mixture of 50 mM sodium phosphate buffer, pH 6.8, and methanol. Detection was by fluorescence (excitation 532 nm, emission 553 nm) with a Hewlett Packard 1046A programmable detector.

### Plasma lipids and other plasma components

The blood samples were sent to a certified medical laboratory (Fürst Medical Laboratory, Oslo, Norway) for lipid and glucose analyses. Serum blood samples were analyzed for total-, HDL- and LDL-cholesterol, triacylglycerols (TAG), and glucose using automated photometric techniques (Modular P Roche; Roche Diagnostics). Plasma LDL-cholesterol was calculated using the Friedwald formula [19]. Plasma fibrinopeptide A (FPA) concentration was measured by competitive ELISA and plasma C-reactive protein (CRP) concentration using a semi-quantitative latex agglutination assay, as described [20,21].

### DNA damage measured in mononuclear cells

The comet assay (single cell gel electrophoresis) [22] was used to measure DNA strand breaks. Mononuclear cells were embedded in low melting point agarose (1%, 75 μL) on microscope slides. Gels were allowed to set at 4°C. Cells were lysed for 1h in 2.5 M NaCl, 0.1 M Na_2_EDTA, 10 mM Tris-HCl, pH 10, 1% Triton X-100 at 4°C to remove membranes, cytoplasm and most nuclear proteins, leaving DNA as nucleoids. To measure strand breaks, the slides were then immersed in elctrophoresis solution (0.3 M NaOH, 1 mM Na_2_EDTA) for 40 min at 4°C and then electrophoresed at 0.8 V/cm for 30 min at an ambient temperature of 4°C. DNA loops containing breaks extend under electrophoresis to form 'comet tails', and the relative intensity of DNA in the tail indicates the DNA break frequency. After neutralisation, gels were stained with 4`,6-diamidine-2`-phenylindole dihydrocloride (DAPI) and viewed by fluorescence microscopy. Tail intensity was assessed by visual scoring; 100 comets selected at random were graded according to degree of damage into five classes (0-4) to give an overall score for each gel of between 0 and 400 arbitrary units [22]).

In addition to frank DNA strand breaks, oxidised bases were measured by incubating, after lysis, with endonuclease III (to detect oxidised pyrimidines) or formamidopyrimidine DNA glycosylase (FPG, recognising 8-oxoGua and other oxidised purines) in 40 mM HEPES, 0.1 M KCl, 0.5 mM EDTA, 0.2 mg/ml bovine serum albumin (pH adjusted to 8.0) for 30 min at 37°C. Net enzyme-sensitive sites were calculated by subtracting the comet score after incubation with buffer alone from the score with enzyme. Due to a comet assay calibration problem in the first intervention period with respect to assessment of endonuclease III sensitive sites, we choose to base all our endonuclease III calculations on samples from the second intervention period. This reduced the effective sample size of endonuclease III sensitive sites from 24 to 12.

### In vitro assay for DNA repair capacity

In this assay, a mononuclear cell extract is incubated with DNA substrate containing specific damage; either 8-oxoGua (to measure BER), or cyclobutane pyrimidine dimers (to measure NER) [22]. HeLa cells were used to prepare substrate for the BER assay by incubating them with 1 μM Ro 19-8022 (gift from F. Hoffmann-La Roche) and irradiating on ice with visible light (5 min at 30 cm from a 500 W tungsten halogen source). As substrate for the NER assay, HeLa cells were irradiated on ice with 1 Jm^2 ^ultraviolet light (UVC). Substrate cells were embedded in agarose and lysed as in the standard comet assay (above). To a thawed aliquot of snap-frozen lymphocytes, 1% Triton X-100 was added to a final concentration of 0.2%, the lysate was centrifuged (15,000 × g, 5 min, 4°C) and the supernatant mixed with 4 volumes of the endonuclease III/FPG incubation buffer (see above) - with addition of 1.6 mM MgCl_2 _in the case of the NER assay [23]. Forty five μL of extract was added to the agarose gel containing substrate nucleoids, incubated for 10 min (BER) or 30 min (NER) and then processed as in the standard comet assay. The production of DNA breaks during the incubation indicates the ability of the extract to carry out the the initial stage of NER. One repair extract was destroyed accidentally in group 2, reducing the effective size of DNA repair samples to 23.

### Gene expression analysis

The blood was collected in Tempus™ blood RNA tubes (4342792, Applied Biosystems). Tempus™ spin RNA isolation kit (4380204, Applied Biosystems) was used with the optional DNase treatment wash buffer (4305545, Applied Biosystems). The extracted RNA was quantified spectophotometrically (NanoDrop) and stored at -80°C. The RNA integrity was assessed on Experion (Bio-Rad) and all samples included in the analysis had RQI values higher than 8. The RNA concentrations were normalized before reverse transcription with the Transcriptor First Strand Synthesis Kit (04896866001, Roche Diagnostics). 5 ng cDNA was added to each PCR reaction and the qPCR was performed using assays from DNA Damage Response Gene Panel (G101, TATAA Biocenter AB) with primer concentrations of 300 nM. The PCR program consisted of 95°C for 3 min, 40 cycles of 95°C for 10 sec, 60°C for 10 sec and 72°C for 10 sec followed by a melt curve with a predenaturation step at 95°C and a melting range of 65-95°C. The PCR was performed on LightCycler^® ^480 (Roche Diagnostics) using PerfeCTa^® ^SYBR^® ^green SuperMix, Low Rox™ (95056-02K, Quanta Biosciences) and VisiBlue™ qPCR mix colorant (K101, TATAA Biocenter AB). Assays for 12 candidate reference genes from the Human endogenous control gene panel (A101, TATAA Biocenter AB) were analyzed for all samples. The two most stable (beta-actin and beta-glucuronidase) were selected using GenEx software (MultiD Analyses AB) and used for normalization. Matched samples of RNA from blood collected before and after supplementation were analysed by quantitative RT-PCR using probes to genes for DNA repair and related processes. The number of paired samples available for gene expression studies was limited to seven.

### Whole blood platelet aggregation

A Chronolog whole blood aggregometer was used to measure the electrical resistance (impedance) to the passage of small electric current across two platinum electrodes immersed in the sample of blood. Platelet aggregation increases impedance. For whole blood aggregation, blood (5 ml) mixed with sodium citrate (final concentration 13 mM) was kept at room temperature for 15 min. Typically, 0.5 mL of blood and 0.5 mL of PBS were placed in a plastic cuvette at 37°C. ADP and collagen at various concentrations were used to initiate the aggregation response [16,24,25]. Due to a technical problem with the aggrometer electrode results from whole blood aggregation measurements are based exclusively on the second intervention period.

### Statistical analysis

Results are presented as the mean ± SEM. Student's t tests were used to test for differences before and after treatment. Results from intervention groups were combined so that data for all subjects receiving one kiwifruit per day, whether in the first or second period, were analysed together, and similarly all data for two per day. Values were considered to be significantly different when p < 0.05. Simple regression analysis was performed to evaluate correlation between plasma vitamin C and strand breaks.

## Results

Both reduced ascorbic acid and total (reduced plus dehydro-) ascorbic acid increased after supplementation with kiwifruit (Table 1), reduced ascorbic acid being borderline significant (p = 0.054) and total ascorbic acid approaching statistical significance (p = 0.068) at 2 kiwifruits per day. No differences were seen between pre- and post-intervention plasma levels of the carotenoids or FRAP values (Table 1). There were no changes in plasma MDA as a result of kiwifruit supplementation (Table 1).

**Table 1:** Plasma antioxidants, FRAP and MDA measured before and after kiwifruit intervention (n = 24)

	Fruits	Before		After		Diff		Change	*p*
		Mean SEM		Mean SEM		Mean SEM		(%)	
		(μmol/L)		(μmol/L)		(μmol/L)			
									
Total ascorbic acid	1	57.11	15.6	59.58	10.9	2.5	19.0	4	0.53
	2	52.83	13.3	60.27	14.3	7.4	19.5	14	0.07
Ascorbic acid, reduced	1	52.79	14.7	55.66	11.2	2.9	18.5	5	0.45
	2	47.97	13	55.43	13.1	7.5	18.5	16	0.05
Lutein, μmol/L	1	0.22	0.1	0.22	0.1	0.0	0.1	0	0.95
	2	0.22	0.1	0.23	0.1	0.0	0.1	5	0.70
β-Cryptoxanthin, μmol/L	1	0.32	0.3	0.31	0.3	0.0	0.4	-3	0.92
	2	0.31	0.3	0.31	0.3	0.0	0.4	0	0.99
α-Carotene, μmol/L	1	0.14	0.1	0.14	0.1	0.0	0.1	0	0.91
	2	0.14	0.1	0.14	0.1	0.0	0.1	0	0.98
β-Carotene, μmol/L	1	0.55	0.3	0.56	0.4	0.0	0.5	2	0.89
	2	0.55	0.3	0.56	0.3	0.0	0.4	2	0.97
Zeaxanthin, μmol/L	1	0.05	0	0.04	0	0.0	0.0	-20	0.91
	2	0.04	0	0.04	0	0.0	0.0	0	0.87
Lycopene, μmol/L	1	0.57	0.1	0.57	0.1	0.0	0.1	0	0.99
	2	0.57	0.2	0.56	0.1	0.0	0.2	-2	0.97
FRAP^1^, μmol/L	1	1203.17	208	1201.13	228.5	-2.0	309.0	0	0.97
	2	1189.38	243.9	1260.46	164.4	71.1	294.1	6	0.24
MDA^2^, μmol/L	1	0.88	0.4	0.88	0.3	0.0	0.5	0	0.97
	2	0.89	0.5	0.88	0.5	0.0	0.7	-1	0.53

SEM: standard error of the mean. ^1^Ferric reducing activity of plasma. ^2^Malondialdehyde.

Table 2 shows plasma concentrations of total cholesterol, HDL, LDL, triglycerides and glucose. Mean glucose, cholesterol, LDL, and HDL cholesterol values were unchanged over the 4-week intervention periods, whereas triglyceride concentrations were significantly lower (by approximately 13%) after supplementation with 1 or 2 kiwifruits per day. Plasma concentrations of FPA over 6 ng/ml were assumed to be the result of unsatisfactory venepuncture and excluded from data analysis; this applied to 4 out of 180 samples. CRP has been shown to induce cytokine imbalance which affects many aspects of platelet function. Base levels of hsCRP (CRP measured with a high sensitivity assay) were 2.34 ± 0.05 mg/L, and were not affected by consumption of kiwifruit.

**Table 2:** Effects of kiwifruit consumption on fasting plasma levels of lipids and glucose (n = 24)

	Before		After		Diff		Change	*p*
	Mean	SEM	Mean	SEM	Mean	SEM	(%)	
	(mM)		(mM)		(mM)			
								
*One kwifruit per day*								
Glucose	4.79	0.1	4.69	0.1	-0.10	0.1	-2.1	0.41
HDL	1.53	0.1	1.52	0.1	-0.01	0.1	-6.5	0.71
LDL	2.32	0.1	2.20	0.1	-0.12	0.1	-3.4	0.68
Total cholesterol	4.05	0.1	3.83	0.1	-0.22	0.2	-4.4	0.26
Triglycerides	0.88	0.1	0.76	0.1	-0.12	0.1	-13.3	0.05
								
*Two kwifruits per day*								
Glucose	4.68	0.1	4.80	0.1	0.12	0.1	2.6	0.20
HDL	1.50	0.1	1.43	0.1	-0.07	0.1	-7.1	0.26
LDL	2.39	0.1	2.51	0.1	0.12	0.1	3.3	0.57
Total cholesterols	4.05	0.1	4.10	0.1	0.05	0.2	4.7	0.51
Triglycerides	0.86	0.1	0.76	0.0	-0.10	0.1	-7.4	0.05

SEM: standard error of the mean

Kiwifruit supplementation, both one and two per day, significantly decreased H_2_O_2_-induced DNA damage (Table 3). Following 4 weeks of golden kiwifruit intervention, a decrease in FPG-sensitive sites in lymphocyte DNA was observed (Table 3), though the decrease was statistically significant only after one kiwifruit per day. Decreases in endonuclease III-sensitive sites were significant after 2 kiwifruits per day but not after one per day (Table 3). In the *in vitro *DNA repair assays, extract prepared from lymphocytes is incubated with specifically damaged DNA containing 8-oxoGua for assessing BER, and cyclobutane pyrimidine dimers for NER. No change in either BER or NER activity was observed following supplementation (Table 3). At two fruits per day there was a borderline significant inverse correlation between plasma levels of reduced vitamin C and lymphocyte strandbreaks (Pearson's *r *= -0.55, p = 0.066).

**Table 3:** Endogenous DNA damage and DNA repair activity in lymphocytes, before and after kiwifruit intervention; Arbitrary units (n = 24)

Fruits		Before		After		Diff		Differ (%)	*p*
		Mean	SEM	Mean	SEM	Mean	SEM		
									
FPG-sensitive sites	1	196.38	73.7	130.33	85.3	-66.05	112.7	-34	0.01
	2	188.58	61.5	148.26	106.7	-40.32	123.2	-21	0.12
Endonuclease III-sensitive sites^1^	1	139.42	71.1	104.00	59.8	-35.42	92.9	-25	0.20
	2	138.17	41.2	92.25	62.1	-45.92	74.5	-33	0.04
Resistance towards H_2_O_2 _oxidation	1	283.08	43.9	189.25	117.2	-93.83	125.2	-33	0.00
	2	278.00	51.6	191.52	124.5	-86.48	134.8	-31	0.00
Base exision repair (BER)	1	48.43	16.3	46.61	14.5	-1.82	21.8	-4	0.69
	2	52.54	13.8	49.11	15.1	-3.43	20.5	-7	0.43
Nucleotide excision repair (NER)^2^	1	39.38	21.3	38.13	23.9	-1.25	32.0	-3	0.85
	2	37.71	18.8	35.50	23.3	-2.21	29.9	-6	0.72

SEM: standard error of the mean. ^1^Based on samples exclusively from the second intervention period (n = 12). ^2^n = 23.

No differences in the expression of any of the genes studied were observed before and after kiwifruit consumption (Table 4).

**Table 4:** Expression of essential DNA repair genes in seven randomly selected subjects during kiwifruit consumption and after wash-out

	Before		After		Diff		Change	*p*
	Mean	SEM	Mean	SEM	Mean	SEM	(%)	
								
Cyclin-dependent kinase inhibitor 1A (p21)	5.32	0.1	5.28	0.2	-0.04	0.2	-0.8	0.80
Replication protein A1 (RPA1)	6.15	0.1	6.31	0.1	0.16	0.2	2.6	0.28
Xeroderma pigmentosum, complementation group A (XPA)	5.56	0.2	5.38	0.2	-0.18	0.2	-3.2	0.30
Excision reapir cross-complementing protein 1 (ERCC1)	3.55	0.1	3.48	0.2	-0.07	0.2	-2.0	0.67
Excision reapir cross-complementing protein 2 (ERCC2)	4.87	0.1	4.79	0.3	-0.08	0.3	-1.6	0.77
8-oxoguanine DNA glycosylas (OGG1)	4.83	0.1	4.83	0.1	0	0.2	0.0	1.00
Proliferating cell nuclear antigen (PCNA)	5.06	0.2	5.01	0.2	-0.05	0.2	-1.0	0.75
X-ray repair cross-complementing protein (XRCC1)	4.07	0.2	3.92	0.1	-0.15	0.2	-3.7	0.41
NAD(P)H dehydrogenase, quinone 1 (NQO1)	7.40	0.1	7.32	0.2	-0.08	0.2	-1.1	0.54
Nuclease (multifunctional DNA repair enzyme) 1 (APEX1)	3.08	0.1	3.00	0.2	-0.08	0.2	-2.6	0.52
Homolog recombination protein (RecA homolog, E. coli)(RAD51)	11.68	0.3	11.58	0.2	-0.1	0.3	-0.9	0.81
Xeroderma pigmentosum, complementation group C (XPC)	4.49	0.1	4.59	0.2	0.1	0.2	2.2	0.58

SEM: standard error of the mean.

Table 5 shows the whole blood platelet aggregation response to different concentrations of ADP and collagen before and after daily consumption of kiwifruit. Whole blood platelet aggregation induced by ADP was reduced at both concentrations after consuming 1 kiwifruit per day; however significance was attained only in the case of 5.0 μM ADP. We observed after 1 kiwifruit per day a significant reduction in whole blood platelet aggregation in response to collagen at 2 μg/ml. Consuming 2 kiwifruits per day had no significant effect on whole blood platelet aggregation.

**Table 5:** Effects of daily consumption of one and two golden kiwifruits per day on whole blood aggregation (n = 24)

Aggregating agent	Before		After		Diff		Change	*p*
	Mean	SEM	Mean	SEM	Mean	SEM	(%)	
	(Resistance, Ohm)		(Resistance, Ohm)		(Resistance, Ohm)			
								
*One kiwifruit per day*								
ADP (5.0 μM)	13.00	2.7	11.40	1.95	-1.6	3.3	-12.3	0.03
ADP (7.5 μM)	13.04	2.0	11.88	2.78	-1.2	3.4	-8.9	0.08
Collagen (1 μg/ml)	16.00	2.1	15.60	2.46	-0.4	3.3	-2.5	0.53
Collagen (2 μg/ml)	18.75	2.2	16.18	1.93	-2.6	2.9	-13.7	0.00
								
*Two kiwifruits per day*								
ADP (5.0 μM)	13.04	3.4	12.17	3.34	-0.9	4.7	-6.7	0.32
ADP (7.5 μM)	12.42	2.7	11.50	2.29	-0.9	3.5	-7.4	0.07
Collagen (1 μg/ml)	15.77	2.4	15.15	1.59	-0.6	2.8	-3.9	0.35
Collagen (2 μg/ml)	17.77	3.4	17.70	2.15	-0.1	4.0	-0.4	0.93

SEM: standard error of the mean.

*In vitro *whole blood platelet aggregation was measured in the presence of undiluted green or golden kiwifruit extract. At similar wet weight the green fruit extract was almost twice as effective as golden kiwifruit extract at inhibiting ADP-induced whole blood platelet aggregation. In contrast, golden kiwifruit extract was significantly more effective than green kiwifruit extract at inhibiting collagen-induced platelet aggregation (data not shown).

## Discussion

Here we report increases in plasma vitamin C and increases in total antioxidant capacity (as indicated by H_2_O_2_-induced DNA damage) as well as a trend towards decrease in oxidised pyrimidines and oxidised purines. Previously, we reported increases in mean plasma vitamin C concentrations of up to 26% after suplementation with 3 green kiwifruits per day for 3 weeks [26]. There were no restrictions in either study as to when during the day the fruit should be eaten, and in practice the last fruit was consumed 10 h or more prior to blood sampling. Peak plasma vitamin C concentrations are reached a few hours after supplementation and then decline [27], and so the 14-16% increase in vitamin C seen here is all the more impressive. Measured a short time after kiwifruit consumption, the concentration would presumably have been much higher. This increase in plasma concentration of reduced and total ascorbic acid indicates that (golden) kiwifruit may be an effective booster of antioxidant status, especially since vitamin C activity is likely to be enhanced by acting in concert with other phytochemicals from fruit.

After both one and two fruits per day resistance towards H_2_O_2_-oxidation increased by ~30%. The reduced level of strandbreaks may in part be caused by the increased intake of vitamin C. At two fruits per day there was a borderline significant inverse correlation between plasma levels of reduced vitamin C and lymphocyte strandbreaks, but this association was not present at one fruit per day. Resistance towards H_2_O_2_-oxidation seemed to increase somewhat more following consumption of yellow kiwifuit than after consumption of green kiwifruit [26], even if a direct comparison cannot be performed between the two studies.

Supplementation with one kiwifruit per day for four weeks protected lymphocytes against DNA base oxidation, as indicated by a decrease of more than 20% in the number of FPG-sensitive sites measured with the comet assay. This effect was not seen after supplementation with two kiwifruits per day. The lack of a clear dose-response effect is not unprecedented; in our previous study with green kiwifruit [10], protective effects against DNA oxidation did not increase with higher daily doses of fruits. This may represent a saturation effect, if the active ingredient in one kiwifruit is sufficient to exert a maximal protective effect. We presently have no idea of the identity of the active ingredient.

Endonuclease III-sensitive sites were also reduced after supplementation by about one quarter. The reduction was significant after two kiwifruits per day but not after one. There was no significant correlation between endonuclease III-sensitive sites and H_2_O_2_-induced DNA damage or plasma vitamin C, indicating that the reduction in endonuclease III-sensitive sites may be caused by specific modulating compounds of the kiwifruits rather than by its antioxidant content per se.

The lack of any effect of kiwifruit supplementation on lymphocyte DNA repair - either BER or NER - is unexpected, since a stimulation of BER was the most pronounced effect in the previous kiwifruit study [10]. Significant effects of green kiwifruit, and of a high fruit and vegetable diet, on both BER and NER, have been seen in another recent intervention trial [28], and so we can hypothesise that golden kiwifruit may lack specific ingredient(s) that can modulate DNA repair. The lack of significant effects of golden kiwifruit supplementation on the expression of key DNA repair genes is consistent with the lack of effect on repair enzyme activities. Lack of effect of antioxidant rich whole food on repair enzyme activities have been reported previously. Despite reduced DNA damage and increased resistance towards H_2_O_2 _induced strand breaks, Rizo *et al. *reported no effect of broccoli consumption on the expression of DNA repair enzymes [29].

There is increasing evidence that some natural components can reduce levels of specific CVD risk factors by modulating platelet function [11,30]. In recent years, research groups including ours have reported inhibitory effects of different fruits on platelet aggregation [11]. Potent anti-platelet-aggregation factor(s) are present in tomatoes and green kiwifruits [11,24,25]. Extracts of these fruits inhibit platelet aggregation in response to ADP, collagen, and thrombin both *in vitro *and *in vivo *[11,21,25].

In the present study, consuming 1 golden kiwifruit per day for 4 weeks reduced whole blood platelet aggregation. This effect disappeared during the washout period (data not shown). Consuming 2 golden kiwifruits generally did not inhibit whole blood platelet aggregation. (Only with 7.5 μM ADP was there a marginally significant decrease.) There is no obvious explanation for this lack of effect, but it corresponds to the lack of dose response in relation to markers of DNA oxidation, discussed above - though there is no reason to assume the operation of a common molecular mechanism. The lack of effects of 2 golden kiwifruits per day could be caused by unknown confounders.

Our *in vitro *platelet aggregation experiments suggest that green and golden kiwifruit extracts inhibit both ADP and collagen-induced whole blood platelet aggregation (to different degrees). The fruit extract may contain a wide variety of different types of compounds that have anti-platelet activity *in vitro *and that affect different mechanisms of activation and aggregation. We have earlier shown that anti-platelet effects of fruits including green kiwifruits and tomatoes are independent of their antioxidant activity [11,16,25].

Consumption of golden kiwifruit reduced plasma triglyceride levels without affecting cholesterol levels: original levels were restored after the washout period (data not shown). Lowering of plasma triglycerides by kiwifruit was observed despite the fact that the volunteers maintained their regular diet during the supplementation period. Such effects of fruits and vegetables have been reported before [31]. Our data imply that both green and golden kiwifruits might have cardio-protective effects.

A possible limitation of the present study was the use of healthy subjects. The protective effect of antioxidant supplementation may be more pronounced in subjects exposed to increased levels of oxidative stress, such as e.g. endurance athletes, diseased or old people [31]. Moderate sample size may be another limitation. Since this was a cross-over intervention any possible weak seasonal variation would cancel out in the final analysis, but the inclusion of a control group could have improved our knowledge of seasonal variations in the measured biomarkers.

To summarise, this intervention trial, with biomarkers relevant to both cancer and CVD indicates that one kiwifruit per day is as effective as two per day, when considering both effects on DNA oxidation, and platelet aggregation. The concentration of plasma triglycerides is decreased by golden kiwifruit, with no parallel effect on cholesterol.

## Conclusion

Regular intake of golden kiwifruit can protect against lymphocyte DNA oxidation as well as increase total antioxidant activity and specifically plasma vitamin C. Regular consumption can also reduce platelet aggregation, and lower plasma triglycerides. Consumption of these fruits could therefore benefit public health by countering oxidative stress factors, and by reducing the risk of thrombotic events mediated by platelet activation.

## Competing interests

The authors declare that they have no competing interests.

## Authors' contributions

AB carried out the intervention study and wrote the initial draft. IG did the NER measurements together with AP. TM and AJ did the blood aggregation measurements. AK measured carotenoids and FRAP in association with RB. JE did the gene expression analysis. AKD and ARC planned the intervention and wrote the manuscript together with AB. All authors read and approved the final manuscript.
